# Development and characterization of microsatellite markers in *Campomanesia adamantium*, a native plant of the Cerrado ecoregions of South America

**DOI:** 10.1002/aps3.11287

**Published:** 2019-09-22

**Authors:** Bruno do Amaral Crispim, Thamiris Gatti Déo, Juliana dos Santos Fernandes, Adrielle Ayumi de Vasconcelos, Maria do Carmo Vieira, Thiago de Oliveira Carnevali, Miklos Maximiliano Bajay, Maria Imaculada Zucchi, Alexeia Barufatti

**Affiliations:** ^1^ Faculdade de Ciências Exatas e Tecnologia Universidade Federal da Grande Dourados Dourados Mato Grosso do Sul 79804‐970 Brazil; ^2^ Faculdade de Ciências Biológicas e Ambientais Universidade Federal da Grande Dourados Dourados Mato Grosso do Sul 79804‐970 Brazil; ^3^ Faculdade de Ciências Agrárias Universidade Federal da Grande Dourados Dourados Mato Grosso do Sul 79804‐970 Brazil; ^4^ Universidade Estadual de Santa Catarina Laguna Rua Cel. Fernandes Martins, 270 ‐ Progresso– Laguna Santa Catarina 88.790‐000 Brazil; ^5^ Agência Paulista de Tecnologia dos Agronegócios Pólo Centro‐Sul (APTA) Piracicaba São Paulo Brazil

**Keywords:** Campomanesia sessiliflora, cross‐amplification, genetic structure, guavira, microsatellite loci, Myrtaceae, simple sequence repeat (SSR) markers

## Abstract

**Premise:**

A novel set of nuclear microsatellite markers was developed and characterized for *Campomanesia adamantium* (Myrtaceae) and tested for cross‐amplification in the related species *C. sessiliflora*.

**Methods and Results:**

Forty‐one primer pairs were designed for simple sequence repeat loci, of which 36 successfully amplified and were polymorphic. The number of alleles ranged from two to 14, with an average of 8.14 alleles per locus. Additionally, cross‐amplification was tested in *C. sessiliflora*; more than 55.5% of the microsatellite loci amplified, confirming the use of these microsatellite markers in a related species.

**Conclusions:**

We developed a set of microsatellite markers that will be useful for future studies of genetic diversity and population structure of *C. adamantium* and a closely related species, which will aid in future conservation efforts.

The Cerrado is the second largest of Brazil's major biomes, after Amazonia, and this biome is characterized by central Brazil's plateau of woodlands, savannas, grasslands, and gallery and dry forests. The climate is seasonal—wet from October to March and dry from April to September—and mild throughout the year, with temperatures ranging from 22°C to 27°C. Average annual rainfall is 1500 mm (Klink and Machado, 2005). The Cerrado biome features a high diversity of native plants with high economic potential for the pharmaceutical and food markets. This biome is a hotspot of biodiversity because endemic species have progressively been threatened by deforestation and agricultural expansion. Research on the genetic and floral biology of these species is extremely important to promote their conservation. Of several native plants of the Cerrado, *Campomanesia adamantium* (Cambess.) O. Berg (Myrtaceae) should be highlighted because of its environmental significance. This species can resist flooding and has been used for spoiled‐area reforestation projects (Hardt et al., 2006). Also known as guavira or gabiroba, *C. adamantium* is commonly found in Brazil and Paraguay and is notable for the potential commercial use of its fruits to produce food and beverages. Many researchers have reported important medicinal properties of this plant, including antioxidant (Coutinho et al., 2008), antimicrobial (Pavan et al., 2009), and antitumor properties (Pascoal et al., 2014).

To start a genetic breeding program for any plant species, it is necessary to assess its genetic variability. Genetic diversity in *C. adamantium* was detected previously using random amplified polymorphic DNA (RAPD) markers (Assis et al., 2013) and by measuring phenotypic characteristics (Resende and Teixeira, 2015). Microsatellite markers have been shown to be efficient tools to assess genetic variation at the individual and population levels because of their hypervariability, wide genomic distribution, co‐dominant inheritance, reproducibility, and multi‐allelic nature (Haq et al., 2014). Recent research has validated the transferability of microsatellite markers from closely related species of *C. adamantium*, including expressed sequence tag (EST)–derived simple sequence repeat (SSR) markers from *Eucalyptus* L'Hér. (Myrtaceae) (Miranda et al., 2016; Crispim et al., 2018) and SSR primers from *Eucalyptus* spp., *Eugenia uniflora* L., and *Melaleuca alternifolia* Cheel (Fagundes et al., 2016). Analysis of genetic diversity using transferable molecular markers only reflects polymorphisms in conserved genomic regions among congeneric species or genera from the same family; however, transferable microsatellite markers from other species may be limited in the level of genetic diversity they can reveal in the target species (Queirós et al., 2015). Thus, the use of species‐specific microsatellites could more accurately report the genetic variability in *C. adamantium* and detect aspects of biodiversity that could be omitted by the use of transferable markers.

Therefore, the goal of this study was to isolate and characterize microsatellite markers for *C. adamantium* by constructing a microsatellite‐enriched genomic library and to test the cross‐amplification of these markers in *C. sessiliflora* (O. Berg) Mattos, a species native to Brazil with medicinal and economic value.

## METHODS AND RESULTS

### Plant material and DNA extraction

Young leaf tissue samples from one accession of *C. adamantium* (voucher 4666, Herbarium of the Federal University of Grande Dourados [DDMS], Dourados, Mato Grosso do Sul, Brazil) were collected to develop the microsatellite markers, and 45 samples from three natural populations were collected to validate the microsatellite markers for this species (Appendix 1). Ten samples of *C. sessiliflora* were collected from a single population to test cross‐amplification of the markers in a related species. Detailed information about all samples collected for this study is provided in Appendix 1. DNA was extracted from young leaf tissue using the cetyltrimethylammonium bromide (CTAB) method (Doyle and Doyle, 1987).

### Development of SSRs and primer design

Genomic DNA of *C. adamantium* was used to develop a microsatellite‐enriched genomic library using a protocol adapted from Billotte et al. (1999). The isolation procedure consisted of digestion of genomic DNA with the *AfaI* restriction enzyme (Invitrogen, Carlsbad, California, USA) and ligation of fragments with double‐stranded adapters 5′‐CTCTTGCTTACGCGTGGACTA‐3′ and 5′‐TAGTCCACGCGTAAGCAAGAGCACA‐3′. The enrichment was based on hybridization capture using (CT)_8_ and (GT)_8_ biotin‐linked probes and streptavidin‐coated magnetic beads (MagneSphere Magnetic Separation Products; Promega Corporation, Madison, Wisconsin, USA). Microsatellite‐enriched DNA fragments were amplified by PCR, cloned into a pGEM‐T Easy Vector (Promega Corporation), and then inserted into *Escherichia coli* XL1‐Blue competent cells (Promega Corporation) using an electroporation technique. Positive clones were selected via a culture medium containing ampicillin and β‐galactosidase.

Ninety‐six colonies were selected using blue/white screening, and the sequencing was performed on an automated ABI 3500xL Genetic Analyzer (Applied Biosystems, Foster City, California, USA) with T7 and SP6 primers and the BigDye Terminator version 3.1 Cycle Sequencing Kit (Perkin Elmer–Applied Biosystems). Microsatellite sequences were identified in 41 clones with good quality sequences for primer design using Primer3Plus software (Untergasser et al., 2012), under the following parameters: size of final amplification products 100–350 bp, GC percentage minimum 40% and maximum 60%, primer annealing temperature ranging from 57°C to 65°C, and the maximum difference in annealing temperature between primer pairs of 3°C.

### Primer validation and microsatellite marker evaluation

Forty‐one primer pairs were designed and tested for amplification in *C. adamantium* in three populations (*n* = 45). In addition, for markers CAMP01, CAMP03, CAMP04, CAMP08, CAMP13, CAMP17, CAMP24, CAMP25, CAMP28, and CAMP36, the genotyping was performed with *n* ≥ 45, using additional individuals sampled from Crispim et al. (2018). Cross‐species amplification was tested using the 36 most polymorphic markers (Table 1). PCR amplifications were performed in a 25‐μL reaction volume containing 50 ng of DNA, 7.5 μL of ultra‐pure water (Fermentas, Waltham, Massachusetts, USA), 0.15 μM of forward and reverse primer, and 12.5 μL of PCR Master Mix (50 U/mL of *Taq* polymerase DNA, 400 μM of dNTP, and 3 mM of MgCl_2_) (Fermentas). PCR cycling conditions consisted of an initial denaturation at 94°C for 5 min; followed by 35 cycles of denaturation at 94°C for 1 min, annealing at 60°C for 1 min, and extension at 72°C for 1 min; and a final extension cycle at 72°C for 15 min. Amplified products were verified visually on 2% agarose gels. Polymorphism evaluation and genotyping were performed using electrophoresis on a 7% polyacrylamide gel stained with silver nitrate (Creste et al., 2001). To determine fragment sizes, we used 10‐bp and 50‐bp DNA ladders (Thermo Fisher Scientific, Waltham, Massachusetts, USA). The fragments were estimated to within 5 bp.

**Table 1:** Characteristics of 36 microsatellite markers developed for *Campomanesia adamantium*

Locus	Primer sequences (5′–3′)	Repeat motif	Allele size range (bp)	GenBank accession no.
CAMP01	F: TATCAAGTCACGAAGGTGGG	(TG)_16_	140–200	MF280931
	R: TGGCAAGTATATCCTGCTCA			
CAMP02	F: CCAATCATGCGATATCGTGC	(TG)_8_	165–210	MF280932
	R: TGGCAAGTATATCCTGCTCA			
CAMP03	F: GTTGGCTCAACAGTTAGCAG	(TG)_8_	150–260	MF280933
	R: TCTAGAACTCGGCATTTCCC			
CAMP04	F: CTTAATGCACATCCGCAACA	(GA)_10_(CA)_6_	200–275	MF280934
	R: GGATGAATTATGTCACGACACA			
CAMP05	F: ACAGAATGTCGTACCTGCAA	(AC)_9_	150–180	MF280935
	R: GGAGTCGAACTGGGTATCTC			
CAMP06	F: GGTTGAGGAACTAGATCGGG	(TG)_17_	225–275	MF280936
	R: GGTGACTTCCGAAACCTTTG			
CAMP07	F: CTCTCTCCTTTCGCATCCTT	(GC)_7_	150–200	MF280937
	R: GCACCTAGTCCCATCAATCA			
CAMP08	F: AATAGCTTCCAGACTGCTCC	(TC)_24_	240–275	MF280938
	R: AAAAGAGAATTTGGAGCGCC			
CAMP09	F: CATCCCGAGTAGCTACAGAG	(AG)_20_	245–270	MF280939
	R: TCACAAACATTGCAAAGGGC			
CAMP10	F: GGAGGGTGACATAAGAGCAA	(TG)_34_	140–195	MF280940
	R: TCCGACTTAACAAGACATACCA			
CAMP11	F: CTGTTCTTGCCACTCTGTTG	(TC)_17_(CA)_16_	200–350	MF280941
	R: ATAATTCCGCCAACAAGCAC			
CAMP12	F: CATATCGCCTTTTTAGCCCG	(TG)_7_	265–270	MF280942
	R: AAAGTGCAGAGAAAGTTCGC			
CAMP13	F: AGTCGAGTGGGCTCTAGTAT	(TG)_8_	200–290	MF280943
	R: ATGTGCTGCTCAGAAAGAGT			
CAMP14	F: TGGTCTTGGTTCCTTTCACA	(GT)_17_	240–300	MF280944
	R: ACTGTGGTAGAGTTTGACCA			
CAMP15	F: CCAATCATGCGATATCGTGC	(TG)_8_	190–230	MF280945
	R: TCACGTGGATTGGCAAGTAT			
CAMP16	F: GAGATCATAGGGCTCTTCGG	(TG)_8_	180–220	MF280946
	R: CCTCGCTAAAGCTTGCTTTT			
CAMP17	F: TCATCTTCGGCTACATAACGT	(AC)_9_	125–160	MF280947
	R: TCCATGCCTTTTCCTCTTTAGA			
CAMP18	F: ACTCGAAGAAGCACTAAGGG	(AC)_10_	190–290	MF280948
	R: TGGTGTTCTGAATTTGGAAGTG			
CAMP19	F: GAGAAGAGAGGGAGCCTTTG	(GA)_28_	160–200	MF280949
	R: CATGGCAACCCTCTTGAATG			
CAMP21	F: GTAGATTGCTGCTAGCTTGC	(TG)_9_	280–315	MF280951
	R: ACCTTCGGTCCCATCAAAAT			
CAMP22	F: TGGTTCCTAAGATCTCCCCA	(TG)_5_	100–180	MF280952
	R: TCCCAAACACTTCTGTATGCT			
CAMP23	F: CCCTTGAAAACTTGTGGTGG	(GA)_27_	150–240	MF280953
	R: CATCTATGTACGAGGGAGGC			
CAMP24	F: CAAGTCCTACATGGCTGGAT	(TG)_7_	200–230	MF280954
	R: AGTGCACGAAAACTGGTCTA			
CAMP25	F: TCCATGCCTTTTCCTCTTTAGA	(TG)_9_	140–180	MF280955
	R: TCATCTTCGGCTACATAACGT			
CAMP26	F: TCTTCGACGAGGTAACAAGG	(AC)_8_	150–215	MF280956
	R: ATGACACACGTGAATACCGA			
CAMP27	F: ATGAAACGGTGGCATCTTTG	(TC)_22_	230–270	MF280957
	R: CGGAAGTTCCATCTCCAAGA			
CAMP28	F: CGTGATGAAGAGTGATGGGA	(GA)_21_	210–240	MF280958
	R: TCATTGATAACTGCGGGTGA			
CAMP30	F: GAGTCCTAGTGCACAATGGT	(TG)_8_(GA)_20_(AG)_5_	270–305	MF280960
	R: TTTTGGGGCTCACTCTATCG			
CAMP33	F: AACTCGTCCAAAATCTCCGT	(GT)_6_	145–240	MF280963
	R: GATTTTGCGTGCTCTCTCTC			
CAMP34	F: TCCGCCCAGACAGAAAATTA	(AC)_8_	180–250	MF280964
	R: AGTTGTCCGACTCCAACTTT			
CAMP36	F: TCCCAAACACTTCTGTATGCT	(CA)_5_	120–170	MF280966
	R: TGGTTCCTAAGATCTCCCCA			
CAMP37	F: GCTCGTAAGGATGTTTTCCC	(GA)_28_	150–220	MF280967
	R: GAGCTTCAACTTGACCAACC			
CAMP38	F: CGATAACCGGCAATATCACG	(TG)_8_(GA)_12_	130–200	MF280968
	R: TCCTCTTTTACCCTCCTCCA			
CAMP39	F: AAAGTAGCTCATGCCCTCTC	(TC)_21_	250–310	MF280969
	R: CTCTGGAATTCGCACGAATC			
CAMP40	F: GCAGCTTAACCACTGGAAAG	(TG)_21_(GA)_8_	270–300	MF280970
	R: TGAAAGCATAGTCGTGTGGA			
CAMP41	F: AGAAAGTGGATGCCGGTTAA	(TG)_8_(GA)_11_	250–310	MF280971
	R: TGGACTTTCACTCACAAGACT			

To evaluate the differences between the genetic diversity parameters of *C. adamantium* resulting from the use of transferable microsatellite markers and specific species, we selected 10 markers developed in this study (CAMP01, CAMP03, CAMP04, CAMP08, CAMP13, CAMP17, CAMP24, CAMP25, CAMP28, CAMP36) and compared them with the data of transferable markers (Embra1335, Embra1076, Embra1470, Embra1364, Embra1363, Embra1374, Embra1811) from the research by Crispim et al. (2018) (Appendix 2).

Statistical analyses were performed using GenAlEx version 6.5 software (Peakall and Smouse, 2012) to calculate the number of alleles per locus, the level of expected heterozygosity (*H*_e_) and observed heterozygosity (*H*_o_), and the polymorphism information content. We used the statistical environment software R (R Core Team, 2018) to estimate the multilocus genotype using the *poppr* R package (Kamvar et al., 2014). Tests for deviation from Hardy–Weinberg equilibrium (HWE) for proportions at each locus for each population were performed using the diveRsity R package (Keenan et al., 2013). The Bonferroni correction was used to correct multiple applications of the same test. We also estimated inbreeding coefficients (*F*_IS_) within each population using the same package. Confidence intervals were obtained with 10,000 bootstrap replicates. The test for null allele presence was performed using MICRO‐CHECKER (van Oosterhout et al., 2004). The influence of null alleles was determined in FREENA (Chapuis and Estoup, 2007) by computing the genetic divergence parameter (*F*_ST_) values using an excluding null alleles (ENA) correction. After accounting for null allele frequencies, loci with frequencies of ≥0.2 were considered potentially problematic for the calculations.

Overall, *C. adamantium* primers successfully amplified 36 SSR loci, in which all markers were polymorphic in the analyzed populations (*n* = 45); the number of alleles ranged from two to 14, with an average of 8.14 per locus. The *H*_o_ and *H*_e_ ranged from 0.00 to 0.91 (average 0.52) and from 0.09 to 0.89 (average 0.78), respectively. When we tested the previously mentioned set of 10 markers (CAMP01, CAMP03, CAMP04, CAMP08, CAMP13, CAMP17, CAMP24, CAMP25, CAMP28, CAMP36) using an increased number of individuals (*n* ≥ 45), the number of alleles ranged from 11 to 23, with an average of 17.10. The level of *H*_o_ and *H*_e_ ranged from 0.24 to 0.72 (average 0.51) and from 0.81 to 0.92 (average 0.87), respectively (Table 2). These results confirm the reliability of the designed set of markers to evaluate genetic diversity in further studies of this species.

**Table 2:** Genetic characterization of 36 microsatellite loci in three populations of *Campomanesia adamantium*.^a^

Locus	Dourados (*n* = 15)					Bonito (*n* = 15)					Cerro Corá (*n* = 15)					Total				
	*A*	*H*_o_	*H*_e_	*F*_IS_ b	HWE^c^	*A*	*H*_o_	*H*_e_	*F*_IS_ b	HWE^c^	*A*	*H*_o_	*H*_e_	*F*_IS_ b	HWE^c^	*n* d	*A*	*H*_o_	*H*_e_	HWE^c^
CAMP01	5	0.42#	0.76	0.45	0.21	7	0.47#	0.77	0.39	0.03	6	0.80	0.72	−0.11	**0.00**	45	12	0.57	0.82	**0.00**
																260	23	0.44	0.91	**0.00**
CAMP02	5	0.36#	0.70	0.49	0.02	8	0.47#	0.82	**0.43**	**0.00**	6	0.73	0.81	**0.10**	0.06	45	11	0.50	0.88	**0.00**
CAMP03	4	0.46	0.68	**0.32**	**0.00**	5	0.47	0.68	**0.31**	0.15	7	1.00	0.78	−0.29	0.22	45	13	0.65	0.87	**0.00**
																240	17	0.63	0.89	**0.00**
CAMP04	5	0.50	0.56	**0.10**	0.25	2	0.71	0.49	−0.46	0.08	4	0.67	0.69	**0.04**	0.57	45	9	0.63	0.77	**0.00**
																275	20	0.72	0.87	**0.00**
CAMP05	5	0.47#	0.72	**0.35**	**0.00**	5	0.87	0.69	−**0.26**	0.15	4	0.67	0.64	−**0.05**	0.03	45	7	0.67	0.79	**0.00**
CAMP06	3	0.00#	0.51	1.00	**0.00**	5	0.73	0.74	**0.01**	**0.00**	6	0.80	0.81	**0.01**	0.02	45	8	0.53	0.85	**0.00**
CAMP07	—	—	—	—	—	3	0.64	0.55	−0.16	0.54	—	—	—	—	—	15	5	0.22	0.73	**0.00**
CAMP08	3	0.33	0.50	**0.33**	0.06	3	0.27	0.24	−0.12	0.95	5	0.93	0.69	−0.36	0.02	45	7	0.52	0.77	**0.00**
																250	20	0.31	0.88	**0.00**
CAMP09	6	0.67	0.73	**0.08**	0.01	3	0.14#	0.36	**0.60**	**0.00**	6	0.73	0.63	−**0.16**	**0.00**	45	8	0.51	0.67	**0.00**
CAMP10	4	0.91	0.67	−0.37	0.16	3	0.67	0.52	−0.29	0.60	5	0.93	0.75	−0.25	0.55	45	10	0.84	0.82	**0.00**
CAMP11	6	0.36#	0.76	0.52	**0.00**	5	0.36#	0.73	0.51	**0.00**	8	0.93	0.81	−**0.15**	0.10	45	14	0.58	0.89	**0.00**
CAMP12	2	0.00	0.17	1.00	**0.00**	—	—	—	—	—	2	0.00	0.12	1.00	**0.00**	30	2	0.00	0.09	**0.00**
CAMP13	8	0.73	0.73	**0.01**	0.10	5	0.25#	0.68	0.63	**0.00**	5	0.57	0.76	**0.25**	0.18	45	11	0.51	0.82	**0.00**
																245	16	0.30	0.91	**0.00**
CAMP14	2	0.00#	0.40	1.00	**0.00**	—	—	—	—	—	6	0.30#	0.76	0.60	0.02	30	7	0.14	0.71	**0.00**
CAMP15	4	0.20#	0.53	0.62	0.01	4	0.58	0.73	**0.20**	**0.00**	3	1.00	0.53	−0.88	**0.00**	45	7	0.65	0.71	**0.00**
CAMP16	3	0.77	0.58	−0.33	0.22	2	0.38	0.45	**0.15**	0.58	4	0.43#	0.69	**0.38**	**0.00**	45	8	0.53	0.83	**0.00**
CAMP17	4	0.73	0.65	−**0.13**	0.01	3	0.33	0.46	**0.28**	0.27	6	0.86	0.82	−**0.05**	**0.00**	45	7	0.64	0.78	**0.00**
																275	14	0.70	0.83	**0.00**
CAMP18	4	0.43	0.64	**0.33**	0.35	8	0.80	0.80	0.01	**0.00**	3	0.53	0.50	−**0.07**	0.39	45	12	0.59	0.82	**0.00**
CAMP19	2	0.00	0.15	1.00	**0.00**	2	0.20	0.42	**0.52**	0.09	4	0.42#	0.73	0.43	0.01	45	5	0.21	0.72	**0.00**
CAMP21	6	0.91	0.79	−0.15	0.45	—	—	—	—	—	—	—	—	—	—	15	6	0.91	0.79	0.45
CAMP22	3	0.69	0.62	−0.11	0.07	—	—	—	—	—	7	0.79	0.73	−0.08	**0.00**	30	10	0.74	0.84	**0.00**
CAMP23	6	0.82	0.74	−**0.11**	0.05	5	0.18#	0.64	0.72	**0.00**	5	0.54	0.66	**0.18**	0.53	45	11	0.51	0.85	**0.00**
CAMP24	2	1.00	0.50	−1.00	**0.00**	2	1.00	0.50	−1.00	**0.00**	2	0.53	0.39	−0.36	0.16	45	2	0.81	0.48	**0.00**
																265	18	0.49	0.81	**0.00**
CAMP25	4	0.82	0.69	−**0.19**	**0.00**	4	0.80	0.57	−0.41	0.35	2	0.36	0.50	**0.27**	0.37	45	6	0.68	0.76	**0.00**
																270	12	0.65	0.81	**0.00**
CAMP26	3	0.21#	0.52	0.59	0.02	2	0.00#	0.50	1.00	**0.00**	3	0.60	0.54	−**0.11**	0.64	45	7	0.29	0.76	**0.00**
CAMP27	4	0.50	0.57	**0.13**	0.61	3	0.30	0.59	**0.49**	0.11	3	0.53	0.66	**0.19**	0.11	45	7	0.46	0.79	**0.00**
CAMP28	4	0.00#	0.48	1.00	**0.00**	3	0.13#	0.55	0.76	**0.00**	4	0.40	0.57	**0.30**	**0.00**	45	7	0.20	0.79	**0.00**
																190	20	0.24	0.92	**0.00**
CAMP30	3	0.55	0.43	−0.26	0.67	2	0.09	0.43	0.79	0.01	4	0.69	0.65	−**0.07**	0.26	45	6	0.46	0.63	0.11
CAMP33	3	0.09#	0.31	0.71	0.01	4	0.85	0.62	−0.37	**0.00**	3	0.62	0.47	−0.31	0.46	45	10	0.54	0.83	**0.00**
CAMP34	4	0.47	0.38	−0.22	0.97	4	0.79	0.59	−**0.33**	**0.00**	3	0.29#	0.57	0.50	**0.00**	45	9	0.51	0.80	**0.00**
CAMP36	4	0.60	0.69	**0.12**	0.04	2	1.00	0.50	−1.00	**0.00**	3	0.87	0.53	−0.65	0.03	45	5	0.85	0.69	**0.00**
																195	11	0.59	0.82	**0.00**
CAMP37	6	0.64	0.65	**0.03**	0.14	5	0.14#	0.68	0.79	**0.00**	5	0.31#	0.72	0.57	**0.00**	45	11	0.34	0.85	**0.00**
CAMP38	5	0.67	0.71	**0.06**	0.01	4	0.60	0.58	−**0.03**	0.73	4	0.42	0.47	**0.10**	0.49	45	10	0.57	0.84	**0.00**
CAMP39	5	0.92	0.78	−**0.18**	**0.00**	4	0.67	0.48	−0.38	0.71	3	0.67	0.58	−**0.15**	0.86	42	9	0.74	0.84	**0.00**
CAMP40	5	0.30#	0.53	0.43	0.40	3	0.29	0.50	0.43	0.18	—	—	—	—	—	30	5	0.29	0.62	**0.00**
CAMP41	8	0.82	0.80	−**0.02**	0.24	3	0.67	0.64	−**0.04**	0.17	3	0.40	0.46	**0.14**	0.80	45	9	0.61	0.80	**0.00**
Average	4.29	0.50	0.59	0.22	0.15	3.84	0.50	0.58	0.13	0.18	4.36	0.62	0.63	0.03	0.21	—	10	0.52	0.78	0.01
**SD**	1.53	0.30	0.17	0.47	0.23	1.65	0.28	0.13	0.51	0.27	1.58	0.24	0.15	0.37	0.26	—	4.81	0.20	0.13	0.07

Note

*A* = number of alleles; *F*_IS_ = inbreeding coefficient; *H*_e_ = expected heterozygosity; *H*_o_ = observed heterozygosity; HWE = Hardy–Weinberg equilibrium; *n* = number of individuals sampled; SD = standard deviation.

a Locality and voucher information are available in Appendix 1.

b Statistically significant deviation based on the 95% confidence interval (data not shown) from the inbreeding coefficient is shown in bold.

c Statistically significant deviation based on Fisher's exact test for Hardy–Weinberg equilibrium after Bonferroni correction (*P* < 0.0001) is shown in bold.

d For markers CAMP01, CAMP04, CAMP08, CAMP13, CAMP17, CAMP24, CAMP25, CAMP28, and CAMP36 genotyping was performed twice: once with *n* = 45 and once with *n* ≥ 45.

^#^ Significant possibility of the presence of null alleles.

Significant deviations from HWE based on Fisher's exact test (*P* < 0.05) in *C. adamantium* were detected for 11 loci in the Dourados population, 15 loci in the Bonito population, and 10 loci in the Cerro Corá population (Table 2). When overall populations were considered, only two markers (CAMP21 and CAMP30) did not significantly deviate from HWE. Several factors (e.g., insufficient sample size, seed dispersal [Hedrick, 2005]) contributed to the observed deviations from HWE. In all sampled regions, *C. adamantium* was observed as a branched tree, very often found in a group of bushes of plants of the same species with the possibility of kinship among sampled individuals; this also was verified by Nucci and Alves‐Junior (2017). Although some individuals with identical multilocus genotypes were found, the observed deviations from HWE may be due to factors such as population subdivision and the presence of null alleles.

The test for null alleles indicated significant results in some loci in the populations (Table 2). The most frequent loci were CAMP01, CAMP02, CAMP11, CAMP14, CAMP26, CAMP28, and CAMP37, which showed a significant possibility of the presence of null alleles in two of the three tested populations. After null allele correction (ENA), overall *F*_ST_ changed only slightly (from 0.302 to 0.283).

In *C. sessiliflora*, 20 microsatellite loci cross‐amplified (Table 3). The number of alleles ranged from two to five, with an average of 2.85. Significant deviations (*P* < 0.05) from HWE were verified in three loci.

**Table 3:** Genetic diversity of 20 microsatellite loci developed in *Campomanesia adamantium* that successfully cross‐amplified in *C. sessiliflora* (*n*=10).^a^^,^ b

Locus	*A*	*H*_o_	*H*_e_	*F*_IS_ c	HWE^d^	Allele size range (bp)
CAMP01	4	0.40	0.66	**0.39**	0.21	160–190
CAMP02	4	0.20	0.48	**0.58**	**0.00**	190–240
CAMP03	2	0.70	0.45	−0.53	0.09	190–200
CAMP04	3	0.80	0.58	−**0.37**	**0.00**	210–300
CAMP05	3	0.10	0.33	0.70	0.01	190–200
CAMP06	2	0.70	0.45	−0.53	0.09	300–310
CAMP09	2	0.20	0.18	−0.11	0.73	250–260
CAMP10	4	0.50	0.56	**0.11**	0.13	175–190
CAMP11	3	0.40	0.48	**0.17**	0.44	180–200
CAMP16	2	0.90	0.49	−0.81	0.01	230–235
CAMP17	2	0.20	0.18	−0.11	0.73	130–140
CAMP23	2	0.80	0.48	−0.66	0.03	150–170
CAMP25	2	0.80	0.48	−0.66	0.03	130–150
CAMP26	3	0.80	0.6	−**0.32**	0.02	240–250
CAMP27	3	0.20	0.33	**0.40**	0.29	210–250
CAMP28	5	0.80	0.74	−**0.08**	0.04	200–240
CAMP33	3	0.30	0.4	**0.25**	0.26	220–260
CAMP36	3	0.20	0.18	−0.08	0.99	140–160
CAMP37	3	0.30	0.58	0.48	0.07	170–210
CAMP39	2	1.00	0.5	−1.00	**0.00**	300–310
Average	2.85	0.52	0.46	−0.11	0.21	
SD	0.88	0.29	0.15	0.49	0.29	

Note

*A* = number of alleles; *F*_IS_ = inbreeding coefficient; *H*_e_ = expected heterozygosity; *H*_o_ = observed heterozygosity; HWE = Hardy–Weinberg equilibrium; *n* = number of individuals sampled.

a Locality and voucher information are available in Appendix 1.

b Cross‐amplification was tested in the 36 polymorphic markers, and results are shown for the 20 markers that successfully cross‐amplified.

c Statistically significant deviation based on the 95% confidence interval (data not shown) from the inbreeding coefficient in bold.

d Statistically significant deviation based on Fisher's exact test for Hardy–Weinberg equilibrium after Bonferroni correction (*P* < 0.002) is shown in bold.

## CONCLUSIONS

We developed a panel of microsatellite markers that will be helpful for future studies of genetic diversity and population structure of *C. adamantium*. The microsatellite loci described in this study successfully cross‐amplified in *C. sessiliflora*, suggesting that these markers could be used to support genetic conservation and breeding programs for *C. adamantium* and other species in the genus.

## AUTHOR CONTRIBUTIONS

B.A.C., A.B., and M.C.V designed the project. T.O.C. collected the samples. B.A.C., A.A.V., T.G.D., J.S.F., and M.M.B performed the experiments. B.A.C., T.G.D., A.B., M.M.B., and M.I.Z. wrote the manuscript.

## Data Availability

Sequence information for the developed primers has been deposited to the National Center for Biotechnology Information (NCBI); GenBank accession numbers are provided in Table 1.

## APPENDIX 1 Voucher information for species used in the development and evaluation of microsatellite markers for *Campomanesia adamantium*.

**Table:**

Taxon	Population	Location	Geographic coordinates	*n*	Voucher no.^b^
*Campomanesia adamantium* (Cambess.) O. Berg^a^	Dourados	Santa Madalena Farm, Dourados, Mato Grosso do Sul, Brazil	22°08′16.3″S, 55°08′24.2″W	15	4666
	Bonito	Bonito, Mato Grosso do Sul, Brazil	21°09’ 02.1″S, 56°28′12.8″W	15	5685
	Cerro Corá	Cerro Corá National Park, Amambay Department, Paraguay	22°39’ 44.2″S, 56°01′52.6″W	15	5686
*Campomanesia sessiliflora* (O. Berg) Mattos	Dourados	Santa Madalena Farm, Dourados, Mato Grosso do Sul, Brazil	22°08’ 25″S, 55°08′17″W	10	5255

Note

*n* = number of individuals sampled.

a The markers CAMP01, CAMP03, CAMP04, CAMP08, CAMP13, CAMP17, CAMP24, CAMP25, CAMP28, and CAMP36 were also tested against additional individuals of *C. adamantium* from Crispim et al. (2018).

b All voucher specimens were deposited in the Herbarium of the Federal University of Grande Dourados (DDMS), Dourados, Mato Grosso do Sul, Brazil.

## APPENDIX 2 Genetic diversity of microsatellites developed in *Campomanesia adamantium* compared to cross‐transferable microsatellites developed in *Eucalyptus*.

**Table:**

Locus	*A*	*H*_o_	*H*_e_	*F*_IS_	HWE
Species‐specific microsatellites (*n* = 124)					
CAMP01	20	0.37	0.89	0.58	0.000*
CAMP03	15	0.70	0.88	0.21	0.000*
CAMP04	14	0.71	0.88	0.18	0.000*
CAMP08	16	0.31	0.83	0.63	0.000*
CAMP13	15	0.25	0.89	0.72	0.000*
CAMP17	14	0.68	0.82	0.17	0.000*
CAMP24	17	0.49	0.89	0.45	0.000*
CAMP25	12	0.64	0.80	0.21	0.000*
CAMP28	28	0.22	0.90	0.75	0.000*
CAMP36	9	0.64	0.83	0.23	0.000*
Average	15	0.50	0.86	0.41	
SD	0.98	0.06	0.01	0.07	
Cross‐transferable microsatellites (*n* = 124)^a^					
Embra1335	4	0.53	0.43	−0.24	0.000*
Embra1076	3	0.98	0.51	−0.94	0.000*
Embra1470	6	0.65	0.56	−0.09	0.000*
Embra1364	20	0.77	0.93	0.15	0.000*
Embra1363	9	0.77	0.83	0.08	0.000*
Embra1374	10	0.67	0.77	0.13	0.000*
Embra1811	8	0.23	0.45	0.49	0.000*
Average	8.57	0.65	0.64	−0.06	
SD	2.15	0.09	0.07	0.17	

Note

*A* = number of alleles per locus; *F*_IS_ = inbreeding coefficient; *H*_e_ = expected heterozygosity; *H*_o_ = observed heterozygosity; HWE = Hardy–Weinberg equilibrium test; *n* = number of individuals sampled.

a Cross‐transferable microsatellite data from Crispim et al. (2018).

* Fisher's exact test significant for Hardy–Weinberg equilibrium proportions after Bonferroni correction (*P* < 0.003).
